# Cyanidin Chloride Induces Apoptosis by Inhibiting NF-κB Signaling through Activation of Nrf2 in Colorectal Cancer Cells

**DOI:** 10.3390/antiox9040285

**Published:** 2020-03-27

**Authors:** Da-Young Lee, Sun-Mi Yun, Moon-Young Song, Kiwon Jung, Eun-Hee Kim

**Affiliations:** College of Pharmacy and Institute of Pharmaceutical Sciences, CHA University, Seongnam, 13488, Korea; angela8804@naver.com (D.-Y.L.); sun21mi@naver.com (S.-M.Y.); wso219@naver.com (M.-Y.S.); pharmj@cha.ac.kr (K.J.)

**Keywords:** cyanidin chloride, Nrf2, NF-κB, apoptosis, colorectal cancer

## Abstract

Colorectal cancer (CRC) is the third most common cancer worldwide and a leading cause of cancer-related deaths in developed countries. Anthocyanins are a class of flavonoids, widely distributed in food, exhibiting important biological effects. Cyanidin chloride (CyCl) is the common type of anthocyanin with antioxidative and anti-inflammatory potential. The present study aimed to investigate the molecular mechanisms underlying the chemotherapeutic effects of CyCl in colorectal cancer cells. We found that CyCl treatment induced apoptosis as well as a significant inhibition of cellular proliferation and colony formation in three colon cancer HCT116, HT29, and SW620 cells. In addition, CyCl suppressed nuclear factor-kappa B (NF-κB) signaling and induced the activation of the nuclear factor erythroid 2-related factor 2 (Nrf2) pathway in tumor necrosis factor-alpha (TNF-α)-stimulated colon cancer cells. Nrf2 and NF-κB are two key transcription factors regulating antioxidative responses and cellular proliferation, respectively. In this study, knockdown of Nrf2 by small interfering RNA (siRNA) transfection inhibited the effect of CyCl on NF-κB signaling and apoptosis, suggesting that there is functional crosstalk between Nrf2 and NF-κB. Our findings demonstrate the important role of Nrf2 in inducing apoptosis through the involvement of NF-κB signaling in colorectal cancer cells, suggesting that CyCl may be used as a potential therapeutic agent for CRC.

## 1. Introduction

Colorectal cancer (CRC) is the third most common cancer worldwide, with estimated annual deaths of more than 500,000. The etiology of CRC varies, but the exact cause is unknown [1]. Several studies have pointed to the nuclear factor-kappa B (NF-κB) signaling pathway as the cause of CRC [2]. NF-κB is a dimer composed of the p50 and p65 subunits and binds to nuclear factor of kappa light polypeptide gene enhancer in B-cell inhibitor (IκB) as a transcription factor present in the cell cytoplasm. When cells are stimulated by pro-inflammatory cytokines, such as tumor necrosis factor-alpha (TNF-α) and interleukin-1 beta (IL-1β), the IκB protein is phosphorylated by the IκB kinases (IKK) complex and NF-κB translocates to the nucleus, increasing the expression of the target genes [3]. Such a substance that increases NF-κB activity induces reactive oxygen species (ROS) formation, and ROS play a central role in regulating the expression of genes involved in the growth, survival, and metastasis of cancer cells by activating NF-κB transcription factors [4]. In particular, the production of excessive ROS in the intestinal lumen causes the cells to expose oxidative stress, thereby inducing cellular damage and contributing to the onset of the disease [5]. 

Cellular ROS levels are regulated by endogenous and exogenous antioxidant systems [6]. Antioxidant or detoxification-related genes have been known to be upregulated by ROS and electrophiles as part of adaptive cellular survival reactions. This concerted response is regulated through a *cis*-acting element known as antioxidant response element (ARE) located in the promoter or enhancer region of various antioxidant genes. The nuclear factor erythroid-derived 2-related factor 2 (Nrf2) has emerged as the important regulator of ARE-dependent transcription [7]. Under normal circumstances, Nrf2 is sequestered in the cytoplasm by an association with the negative regulator Kelch-like ECH-associated protein 1 (Keap1). In the presence of oxidative stress, Nrf2 is released from Keap1 and translocates to the nucleus [8]. The nuclear Nrf2 then interacts with the ARE located in the promoter of genes encoding antioxidant/detoxifying enzymes, such as heme oxygenase-1 (HO-1), glutathione *S*-transferases (GST), NAD(P)H: quinone oxidoreductase 1 (NQO1), etc. [9], and activates their transcription. Most of the experimental evidence suggests that the activation of the Nrf2 pathway confers cytoprotection under various stimulations in many animal models [10]. However, the effect of Nrf2 on apoptosis in cancer cells remains poorly investigated.

Recently, several antioxidants have been reported to block NF-κB activity and participate in the activation of Nrf2 signaling [11,12]. Anthocyanins are water-soluble pigments and are contained in colored fruits and vegetables, such as cherries, grapes, and peaches [13]. It is the secondary metabolites that belong to the polyphenol group named flavonoids [14,15]. In particular, anthocyanins having structural phenol rings have an advantage in free radical scavenging activity [14]. Anthocyanins are the best well-known antioxidants and are positively charged to the oxygen atom of the C-ring of the basic flavonoid structure. Although anthocyanin intake is not essential, studies have reported that anthocyanins and other bioactive compounds improve health throughout the lifetime [15]. In particular, it has been found that the antioxidant effect of anthocyanins not only has a beneficial effect on health but also has a body protective function, such as anti-cancer action, along with several other phytochemicals [13,16]. Cyanidin chloride (CyCl) is the most common type of anthocyanin with red purple natural organic compound [17]. Recent studies have reported that CyCl exhibits inhibitory effects on the growth of colon cancer cells by controlling cell cycle arrest and stress proteins [18], and anti-hyperlipidemic and anti-inflammatory effects in kidney cells [19]. CyCl has also been shown to inhibit nitric oxide production induced by lipopolysaccharide and increase the expression of antioxidant enzymes via Nrf2 activation in murine microglial cells [20]. Based on this evidence, we therefore hypothesized that CyCl may have an anti-cancer effect on colon cancer cells through the induction of apoptosis by inhibition of NF-κB signaling. Moreover, we investigated the role of Nrf2 against CyCl-induced apoptosis and the NF-κB signaling pathway in colon cancer cells. We showed that knockdown of Nrf2 abolished apoptotic responses induced by CyCl as well as the inhibition of the NF-κB signaling pathway. This is the first study demonstrating the role of Nrf2 in inducing apoptosis through the regulation of NF-κB signaling in colon cancer cells. Our results suggest that CyCl may be used as a potential therapeutic agent for CRC.

## 2. Materials and Methods

### 2.1. Cell Culture and Cell Viability Assay

Human colon cancer HCT116, HT29, and SW620 cells were purchased from the American Type Culture Collection (ATCC, Rockville, MD, USA) and maintained in accordance with ATCC’s guidelines. For cell viability assay, cells (1 × 10^6^) were seeded in 6-well plates and were incubated for 24 h, and then media were changed with fresh one containing CyCl (European directorate for the quality of medicines and healthcare, Strasbourg, France). After 24, 48, and 72 h, cells were harvested with trypsinization. Cells were stained with trypan blue and were counted using a hemocytometer. The mean ± SD of at least three independent experiments was displayed.

### 2.2. Flow Cytometry

Flow cytometry was measured using a FITC Annexin V/dead cell apoptosis kit with FITC Annexin V and propidium iodide (PI), for flow cytometry (Invitrogen, Waltham, MA, USA). Briefly, cells (1 × 10^6^) were seeded on 6-well plates and were cultured for 24 h, then treated with 50 and 100 µM of CyCl. After 24 h, cells were washed with cold PBS and were digested by 0.25% ethylenediaminetetraacetic acid (EDTA)-free trypsin. After centrifugation, the supernatant was discarded, and cells were suspended with binding buffer. For each sample, 5 µL of Annexin V and 1 µL of propidium iodide (PI) were added and cells were incubated in room temperature for 15 min. All cells were analyzed by a CytoFLEX flow cytometry system (Beckman Coulter Inc., Brea, CA, USA).

### 2.3. MTT Assay

HCT116 cells (1 × 10^5^) were plated on 96-well plates and were cultured for 24 h, then replaced with fresh medium containing dimethyl sulfoxide (DMSO) or CyCl. After 24 h, the medium was changed to 3-(4,5-dimethylthiazol-2-yl)-2,5-diphenyltetrazolium bromide (MTT) diluted to 1 mg/mL and cultured for 3 h. After 3 h, the media was removed and 50 µL of DMSO was added and measured at 570 nm absorbance.

### 2.4. IKKβ Kinase Activity Measurement

The activity of IKKβ kinase was measured by the SelectScreem™ Biochemical Kinase Profiling Service provided by Thermo Fisher Scientific (Waltham, MA, USA). The Z’-LYTE biochemical assay was analyzed by the differential sensitivity of phosphorylated and non-phosphorylated peptides to proteolytic cleavage with fluorescence-based binding enzyme types. Emission rate = Donor Emission (445 nm)/Acceptor Emission (520 nm).

### 2.5. Luciferase Assay

HCT116 cells were transfected with NF-κB or Nrf2 promoter-driven luciferase reporter plasmid using Lipofectamine^®^ 2000 Transfection Reagent (Invitrogen, Waltham, MA, USA) following the manufacturer’s instructions. At 24 h post-transfection, HCT116 cells were treated with CyCl at the indicated concentration. After the indicated time period, cells were lysed and a luciferase assay was performed using the luciferase assay system (Promega, Madison, WI, USA) in accordance with the manufacturer’s guidelines. Each analysis was performed in triplicate.

### 2.6. 2,2-Diphenyl-1-Picrylhydrazyl (DPPH) Free Radical Scavenging Assay

CyCl was dissolved in DMSO and was adjusted to indicate doses. DPPH solution was prepared. CyCl was added to the DPPH solution, and the mixture was reacted at room temperature for 10 min. Then, the absorbance was measured at 517 nm. The absorbance value obtained after the reaction was used. The scavenging activity of DPPH was calculated as:

scavenging activity (%) = [1 − (A/B)] × 100(1)

where A: absorbance of the sample and B: absorbance of the control.

### 2.7. Focus-Forming Assay

Five hundred cells were seeded per well in 6-well plates. After incubation for 2 weeks with the media changed every 3 days, the colonies were rinsed with phosphate-buffered saline (PBS) and stained with 2% methylene blue. All experiments are carried out in triplicates.

### 2.8. Isolation of Nuclear and Cytoplasmic Fractions

Cytosol and nucleus fractions were isolated using NE-PER nuclear and cytoplasmic extraction reagents (Thermo Scientific, MA, USA) according to the manufacturer’s instructions. Briefly, after washing the cells with DPBS, cells were harvested and centrifuged for 5 min at 3000 rpm to remove the supernatant. Cytoplasmic extraction reagent (CER) I and CER II were added to the pellet, vortexed and incubated for 10 min on ice, and centrifuged for 1 min at 12,000 rpm. The supernatant (cytoplasmic extract) was immediately transferred to a new tube, and the cell pellet was suspended in nuclear extraction reagent. The pellets were incubated on ice for 40 min, vortexed every 10 min, and centrifuge for 10 min at 12,000 rpm. Then, the supernatant was transferred to a new tube and all samples were analyzed by Western blotting.

### 2.9. Western Blot Analysis

The cells were lysed with cell lysis buffer containing protease inhibitor (Roche Applied Science, Mannheim, Germany). The cells were centrifuged for 15 min at 13,000 rpm. Western blot analysis was performed as previously described [21]. The proteins were loaded on to 10% sodium dodecyl sulfate–polyacrylamide gel electrophoresis (SDS-PAGE) and transferred to polyvinylidene fluoride membranes, which were incubated with the primary antibodies (Table 1). Then, membranes were washed and incubated with peroxidase-conjugated secondary antibodies. Membranes were rewashed, and then were visualized using an enhanced chemiluminescence system (Thermo Fisher Scientific, Waltham, MA, USA).

### 2.10. RNA Preparation and Gene Expression Analysis

Total mRNA was isolated from the cells using Trizol reagent (Initrogen, Waltham, MA, USA) and cDNA was prepared using a SuperScript^®^ II Reverse Transcriptase kit (Invitrogen, Waltham, MA, USA) according to the manufacturer’s instructions. The mRNA levels were assessed by quantitative real-time PCR (qRT-PCR) and reverse transcription PCR (RT-PCR). qRT-PCR was performed as previously reported [22] and was assessed on a ViiA^TM^ 7 Real-time PCR system (Applied Biosystems, Foster city, CA, USA) using SYBR premix Ex Taq^TM^ (Takara Bio, Kusatsu, Shiga, Japan). The relative quantities of target genes were calculated from triplicate samples after normalization by an internal control, 18S ribosomal RNA (18s rRNA). RT-PCR was performed as previously described [23] and was measured for 35 cycles at 94 °C for 20 s, 58 °C for 30 s, and 72 °C for 45 s. Oligonucleotide primers are indicated in Table 2.

### 2.11. Small Interfering RNA (siRNA)

The siRNA against Nrf2 was produced from Dharmacon, Inc. (Lafayette, CO, USA). For the gene knock-down experiments, HCT116 cells were seeded onto 6-well plates and siNrf2 was transfected into cells using Lipofectamine^®^ 2000 Transfection Reagent (Invitrogen, Waltham, MA, USA) according to the manufacturer’s protocol. After 24 h, CyCl was treated and cultured for 24 h. Then, 24 h later, cells were washed with DPBS, and were prepared for the next experiment. Target specificity and nonspecific gene silencing effects were measured as a control group treated with negative control siRNA.

### 2.12. Statistical Analysis

Results were expressed as the mean ± standard deviation. The statistical significance was analyzed by one-way analysis of variance (ANOVA). Statistical significance was accepted at *p* < 0.05.

## 3. Results

### 3.1. CyCl Inhibits Cell Proliferation and Induces Apoptosis in Colon Cancer Cells

**Figure 1:**
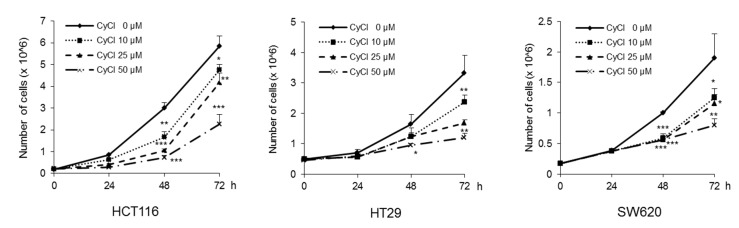
Cyanidin chloride (CyCl) inhibits cell proliferation in colon cancer cell lines. The antitumor effect of CyCl on HCT116, HT29, and SW620 cells was measured by trypan blue staining. Cells were treated with 0, 10, 25, and 50 µM of CyCl for 24, 48, and 72 h. Each experiment was performed in triplicate. * *p* < 0.05, ** *p* < 0.01, and *** *p* < 0.001, significantly different compared with control.

**Figure 2:**
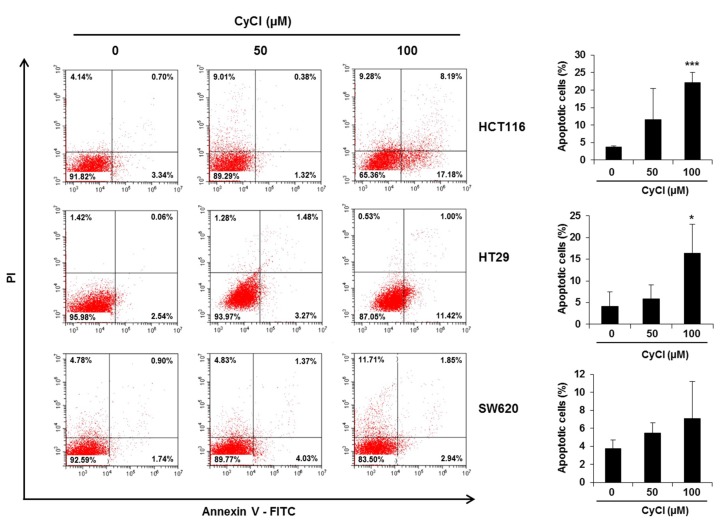
CyCl induces apoptosis in colon cancer cell lines. Cells were treated with 50 and 100 µM of CyCl for 24 h. The apoptotic rates were analyzed by flow cytometry using Annexin V-FITC/PI staining. Each experiment was performed in triplicate. * *p* < 0.05 and *** *p* < 0.001, significantly different compared with control.

**Figure 3:**
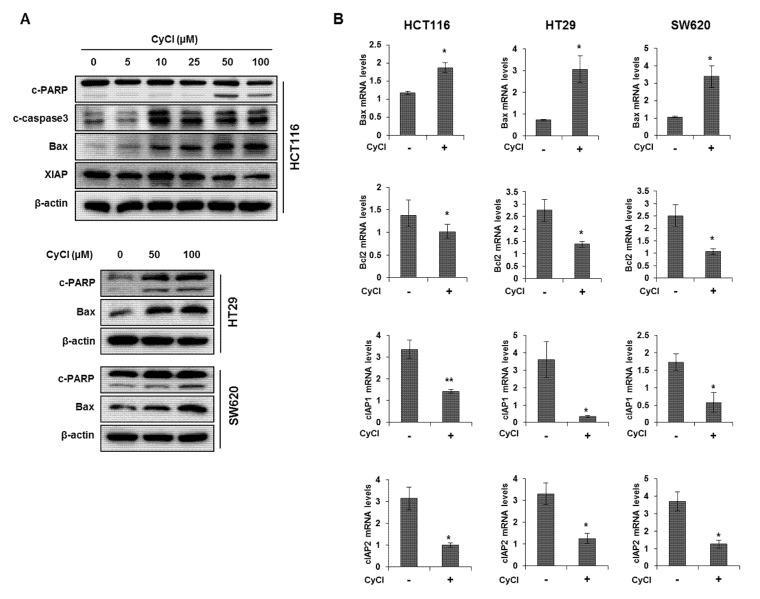
CyCl induces apoptosis in colon cancer cell lines. (**A**) HCT116, HT29, and SW620 cells were treated with indicated concentrations of CyCl and incubated for 24 h. Protein extracts were separated by SDS-PAGE, and Western blot analysis was conducted for the levels of B-cell lymphoma 2-associated X protein (Bax), X-linked inhibitor of apoptosis protein (XIAP), cleaved caspase-3, and poly-ADP-ribose polymerase (PARP) cleavage. (**B**) Colon cancer cells were treated with 50 µM of CyCl and incubated for 24 h. RNA was extracted from the cells and mRNA expression of Bax, B-cell lymphoma 2 (Bcl2), cellular inhibitor of apoptosis protein (cIAP)1, and cIAP2 was measured by qRT-PCR analysis. All experiments were carried out in triplicate. * *p* < 0.05 and ** *p* < 0.01, significantly different compared with control.

To assess the direct inhibitory effect of CyCl on colon cancer cells, three colon cancer cell lines, HCT116, HT29, and SW620, were exposed to various concentrations of CyCl for 72 h and trypan blue staining was employed to measure the changes in cell viability. The CyCl treatment resulted in a significant decrease in cell proliferation in a time- and dose-dependent manner in all colon cancer cells compared to the treatment with DMSO (*p* < 0.05) (Figure 1). To determine whether apoptosis induction contributed to the inhibitory effect of CyCl on cell viability, we analyzed the apoptotic effect of CyCl on three colon cancer HCT116, HT29, and SW620 cells using flow cytometry analysis. As shown in Figure 2, treatment with CyCl significantly induced cell death in a concentration-dependent manner in HCT116 and HT29 cells. These results were further validated by Western blotting analysis of proteins associated with apoptosis, in which treatment with CyCl caused a significant increase in the expression of markers related to the apoptotic process (Figure 3A and Figure S1A). In contrast, the anti-apoptotic protein, X-linked inhibitor of apoptosis protein (XIAP), was significantly reduced by CyCl treatment in HCT116 cells (Figure 3A and Figure S1A). We also measured the expression of mRNA levels related to apoptosis in CyCl-treated colon cancer cells. The mRNA levels of the pro-apoptotic marker B-cell lymphoma 2-associated X protein (Bax) were significantly increased by CyCl treatment, whereas the mRNA levels of anti-apoptotic markers B-cell lymphoma 2 (Bcl2) cellular inhibitor of apoptosis protein (cIAP)-1, and cIAP2 were significantly decreased in CyCl-treated colon cancer cells (Figure 3B and Figure S1B). These findings suggest that CyCl treatment inhibits colon cancer cell proliferation through the triggering of apoptotic responses. 

### 3.2. CyCl Suppresses the NF-κB Signaling Pathway in Colon Cancer Cells

**Figure 4:**
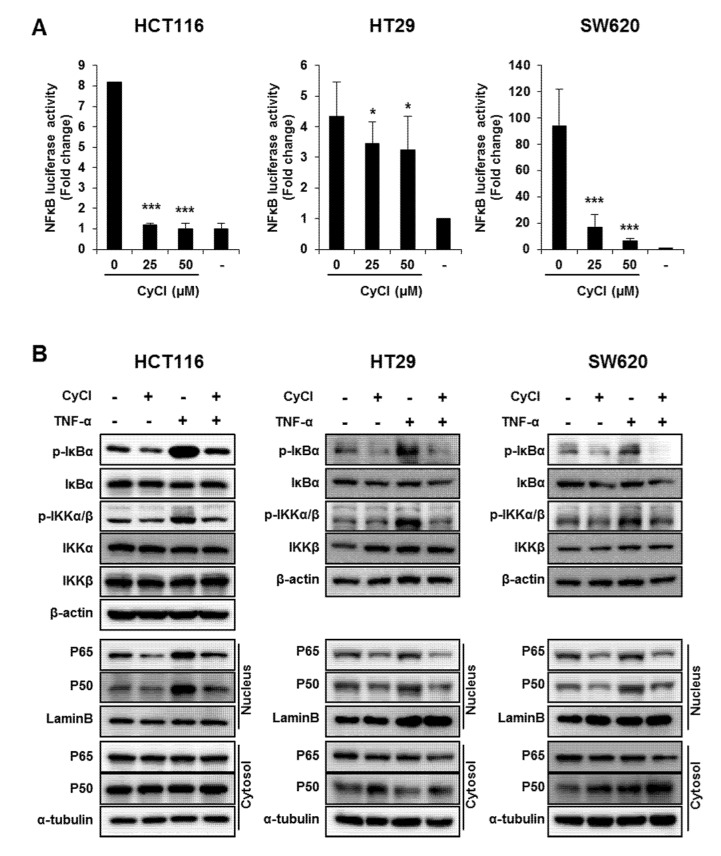

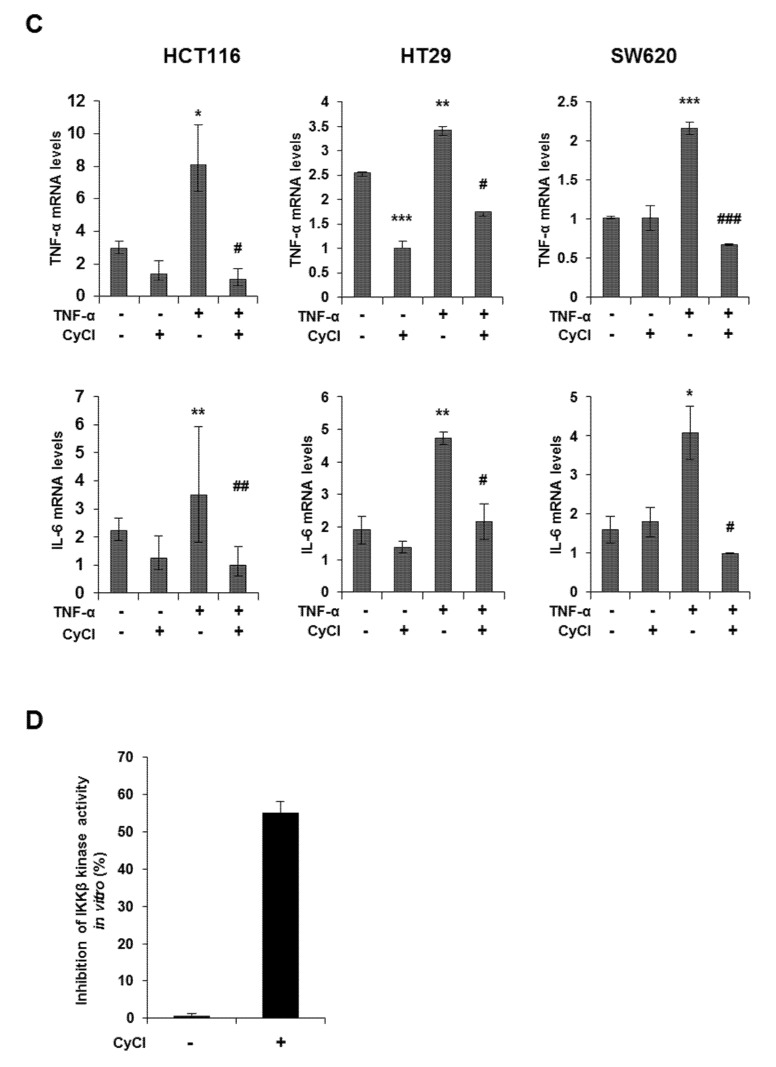
CyCl suppresses the nuclear factor kappa-light-chain-enhancer of activated B cells (NF-κB) signaling pathway in colon cancer cells. (**A**) HCT116, HT29, and SW620 cells were transiently transfected with NF-κB-Luc plasmids for 24 h and treated with 0, 25, and 50 µM of CyCl for 24 h. Cell extracts were harvested, and luciferase assays were performed. (**B**) HCT116, HT29, and SW620 cells were treated with tumor necrosis factor-alpha (TNF-α) and 50 µM of CyCl and incubated for 24 h. Protein extracts were separated by SDS-PAGE, and Western blot analysis was conducted for the expressions of p-inhibitor of kappa B alpha (IκBα), IκBα, p-inhibitor of nuclear factor kappa-B kinase subunit alpha/beta (IKKα/β), IKKα, IKKβ, p65, and p50 protein. (**C**) HCT116, HT29, and SW620 cells were treated with TNF-α and 50 µM of CyCl and incubated for 24 h. RNA was extracted from the cells and mRNA expression of TNF-α and IL-6 was measured by qRT-PCR analysis. (**D**) The inhibitory effect of CyCl (10 μM) on IKKβ kinase activity in vitro was analyzed by the SelectScreen™ Biochemical Kinase Profiling Service. * *p* < 0.05, ** *p* < 0.01, and *** *p* < 0.001, significantly different compared with control; # *p* < 0.05, ## *p* < 0.01, and ### *p* < 0.001, significantly different compared with TNF-α-treated cells.

Since NF-κB is known as a key element in colorectal carcinogenesis, we evaluated the inhibitory effect of CyCl on NF-κB promoter activity in colon cancer cells. As a result, CyCl treatment significantly decreased NF-κB promoter activity in a dose-dependent manner in colon cancer cells (Figure 4A). Moreover, TNF-α-induced phosphorylation of IκBα and IKKα/β, characteristics of canonical NF-κB signal activation, was dramatically inhibited by the treatment with CyCl in colon cancer cells. The nuclear translocation of p65 and p50 was significantly increased in TNF-α-activated colon cancer cells while CyCl treatment abrogated the translocation of these subunits to the nucleus (Figure 4B and Figure S2A). Next, we tested the effect of CyCl on the activation of NF-κB-regulated genes in colon cancer cells. As shown in Figure 4C and Figure S2B, CyCl treatment suppressed the expression of NF-κB target genes, such as NF-κB inhibitor α and δ, TNF-α, IL-6, and IL-8, in TNF-α treated colon cancer cells. To explore the inhibitory effect of CyCl on the NF-κB signaling pathway, in vitro fluorescence-based coupled-enzyme-type IKKβ kinase activity was analyzed, and we observed that the suppressive effect of CyCl on IKKβ kinase activity was 55% compared with the control (Figure 4D).

### 3.3. CyCl Induces Translocation of Nrf2 into the Nucleus and Activates Antioxidant Enzymes

**Figure 5:**
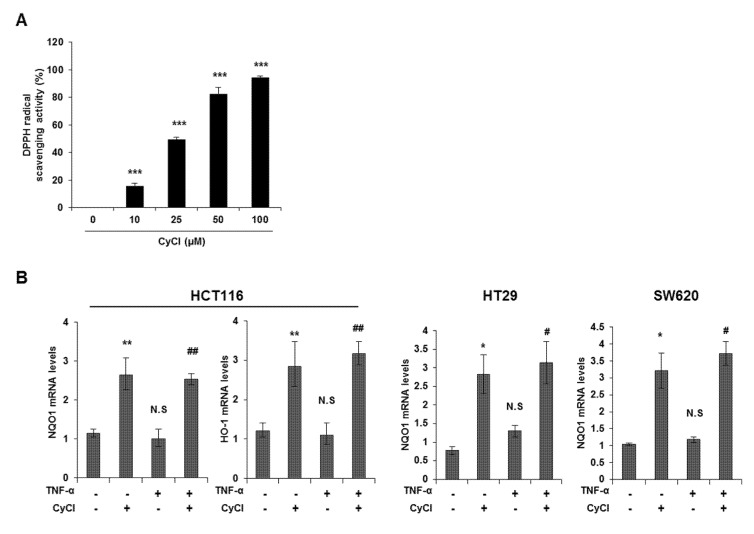

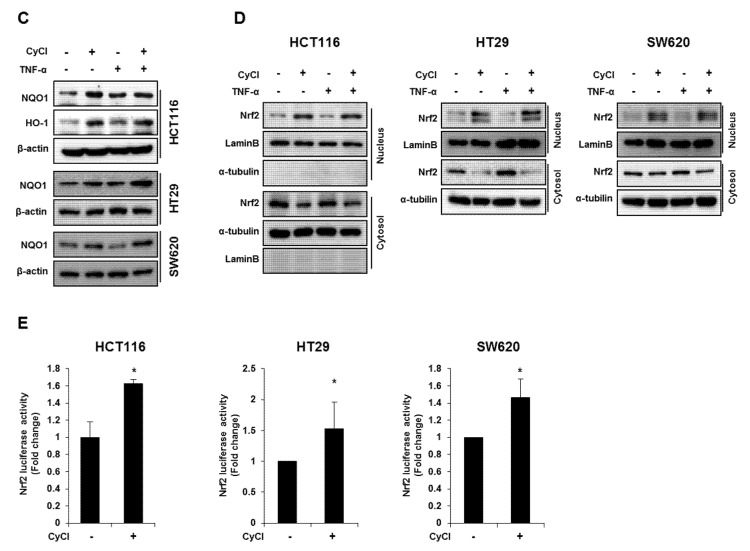
CyCl induces Nrf2 activation and the expression of antioxidant enzymes. (**A**) 2,2-Diphenyl-1-Picrylhydrazyl (DPPH) radical scavenging activity (%) of CyCl. (**B**) HCT116, HT29, and SW620 cells were treated with TNF-α and 50 µM of CyCl and incubated for 24 h. RNA was extracted from the cells and mRNA expression of NAD(P)H quinone dehydrogenase 1 (NQO1) and heme oxygenase-1 (HO-1) was measured by qRT-PCR analysis. (**C**) HCT116, HT29, and SW620 cells were treated with TNF-α and 50 µM of CyCl and incubated for 24 h. Protein extracts were separated by SDS-PAGE and Western blot analysis was conducted for the expression of NQO1 and HO-1 protein. (**D**) Representative immunoblots of nuclear and cytosolic proteins demonstrating changes in the subcellular localization of Nrf2 following CyCl treatment in TNF-α-stimulated cells. The quantitative results are expressed as the mean ± SD of three independent experiments. (**E**) HCT116, HT29, and SW620 cells were transiently transfected with Nrf2-Luc plasmids for 24 h and treated with 50 µM of CyCl for 24 h. Cell extracts were harvested, and luciferase assays were performed. All experiments were carried out in triplicate. * *p* < 0.05, ** *p* < 0.01, and *** *p* < 0.001, significantly different compared with control; # *p* < 0.05 and ## *p* < 0.01, significantly different compared with TNF-α-treated cells.

CyCl is the most common anthocyanin known as a powerful antioxidant because it is sensitive to ROS due to its electron-scavenging ability [17,24]. Consistent with this notion, CyCl treatment significantly increased DPPH radical-scavenging activity in a dose-dependent manner in vitro (Figure 5A). To better define the antioxidant effect of CyCl, we examined the ability of CyCl to modulate the expression of antioxidant enzymes and Nrf2 in TNF-α-stimulated colon cancer cells. As shown in Figure 5B,C and Figure S3A, the addition of CyCl to colon cancer cells resulted in a significant increase in mRNA and protein levels of the expression of antioxidant enzymes with or without TNF-α stimulation. In subsequent experiments, we investigated the effect of CyCl on the expression and transcriptional activity of Nrf2, a known key transcription factor for antioxidant enzymes. The treatment with CyCl significantly induced the nuclear translocation of Nrf2 in colon cancer cells with or without TNF-α stimulation (Figure 5D and Figure S3B). Moreover, Nrf2 luciferase analysis verified that Nrf2 binding to the ARE promoter was significantly increased in CyCl-treated colon cancer cells (Figure 5E), indicating the antioxidative actions of CyCl through activation of the Nrf2 signaling pathway. 

### 3.4. Nrf2 Activation is Crucial for Apoptosis Induced by NF-κB Suppression

**Figure 6:**
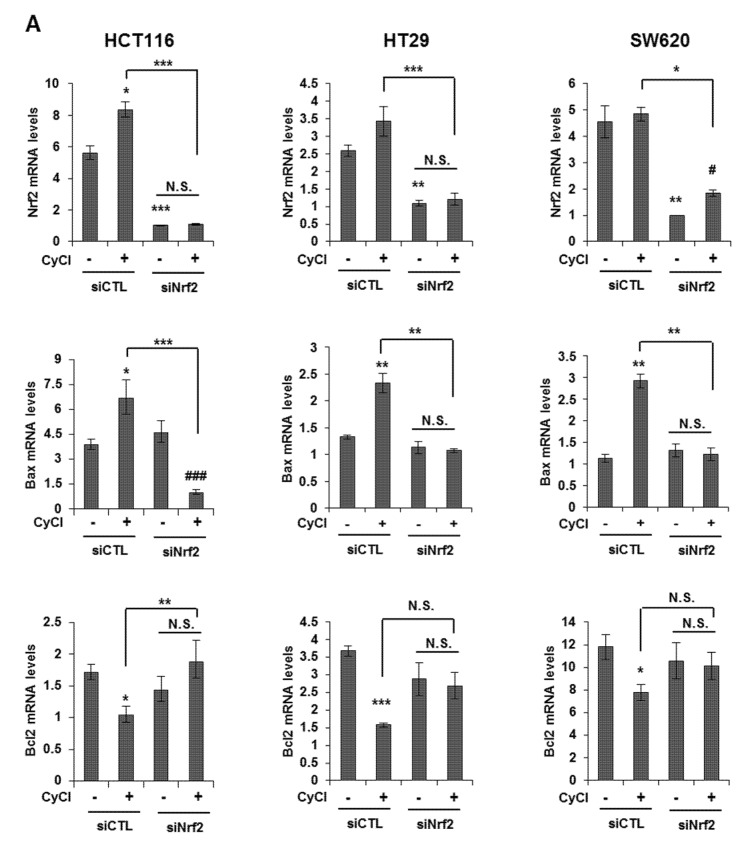

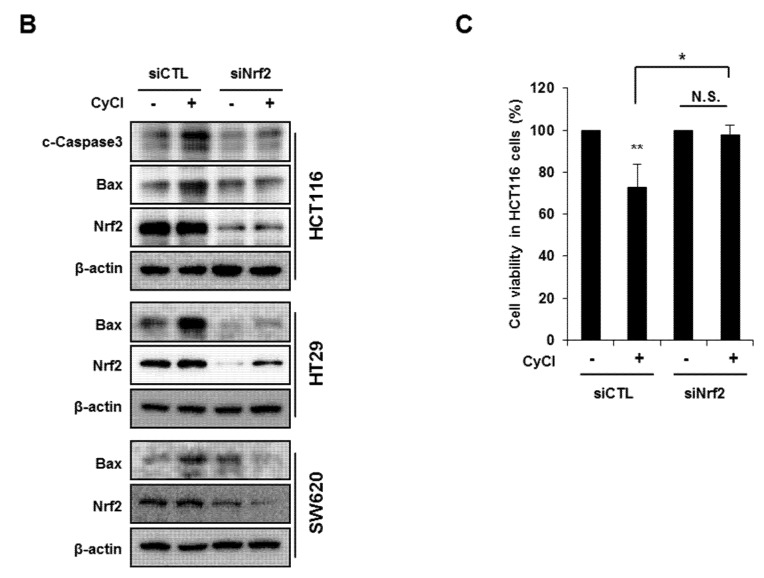
Knockdown of Nrf2 by Nrf2 small interfering RNA (siRNA) abolishes CyCl-induced apoptosis. (**A**) HCT116, HT29, and SW620 cells were transfected with 15 nM of control or Nrf2 siRNA. After 24 h, cells were treated with 50 µM of CyCl for 24 h and harvested. The expression of Nrf2, Bax, and Bcl2 mRNA was measured by qRT-PCR. (**B**) HCT116, HT29, and SW620 cells were transfected with 15 nM of control or Nrf2 siRNA for 24 h and treated with 50 µM of CyCl for 24 h. After protein isolation, Western blot analysis was conducted for the levels of cleaved caspase-3, Bax, and Nrf2 protein. (**C**) HCT116 cells were transfected with control or Nrf2 siRNA for 24 h and treated with 50 µM of CyCl for 24 h. Cell viability was evaluated by 3-(4,5-dimethylthiazol-2-yl)-2,5-diphenyltetrazolium bromide (MTT) assay. All experiments were carried out in triplicate. * *p* < 0.05, ** *p* < 0.01, and *** *p* < 0.001, significantly different compared with empty vector-transfected control; # *p* < 0.05 and ### *p* < 0.001, significantly different compared with siNrf2-transfected control.

Multiple lines of evidence indicate that the potential crosstalk between the Nrf2 and NF-κB pathway in various experimental models [25,26]. Nrf2 is known as an important anti-cancer factor and a major regulator for cancer chemoprevention [26]. To explore the relationship of Nrf2 and apoptosis in CyCl-treated colon cancer cells, we examined the effect of Nrf2 inhibition by siRNA transfection on apoptosis induced by CyCl treatment. RT-PCR and qRT-PCR results showed that the expression of Nrf2 is inhibited by Nrf2 siRNA transfection (Figure S4A and Figure 6A). As shown in Figure 6A, knockdown of Nrf2 resulted in decreased mRNA expression of Bax and increased mRNA expression of Bcl2. We next examined the effect of Nrf2 knockdown on the apoptotic process. Transfection with Nrf2 siRNA indeed abolished the up regulation of apoptotic markers induced by CyCl treatment in colon cancer cells (Figure 6B and Figure S4B), suggesting the role of Nrf2 in the regulation of apoptosis induced by CyCl. Moreover, we observed that knockdown of Nrf2 abrogated colon cancer cell death induced by CyCl in HCT116 cells transfected with Nrf2 siRNA (Figure 6C). These results suggest that the activation of Nrf2 by CyCl treatment led to the activation of downstream apoptotic signaling pathways, which in turn contributed to the anti-colon cancer activity of CyCl.

**Figure 7:**
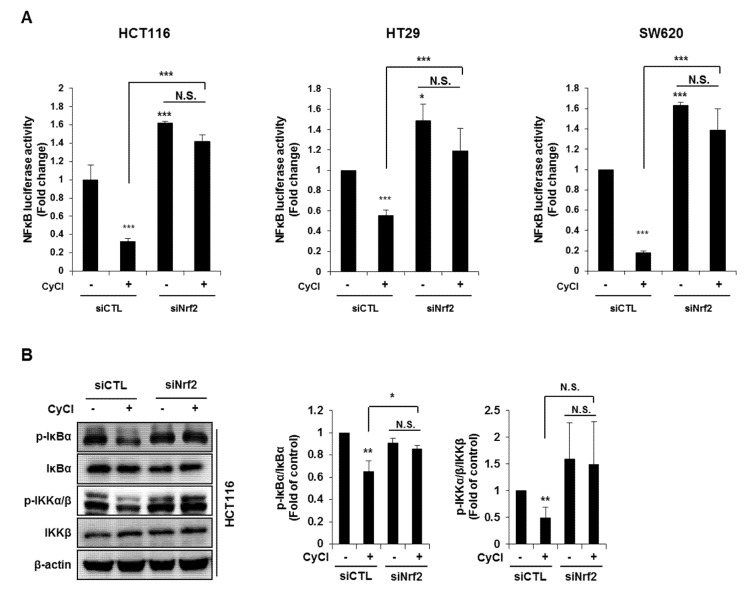
Nrf2 knockdown abrogates the suppression of NF-κB signaling induced by CyCl. (**A**) The NF-κB-response-element-Luc reporter was cotransfected with Nrf2 siRNA into colon cancer HCT116, HT29, and SW620 cells. After 24 h, cells were treated with or without 50 µM of CyCl. After 24 h, cells were lysed for the luciferase assay. (**B**) HCT116 cells were transfected with control or Nrf2 siRNA. After 24 h, cells were treated with 50 µM of CyCl for 24 h. Western blot analysis was performed for the levels of p-IκBα, IκBα, p-IKKα/β, and IKKβ protein. All experiments were carried out in triplicate. * *p* < 0.05, ** *p* < 0.01, and *** *p* < 0.001, significantly different compared with empty vector-transfected control.

NF-κB is an active player in cancer cell proliferation, which allowed us to evaluate the effect of Nrf2 knockdown on the NF-κB signaling pathway in CyCl-treated colon cancer cells. As mentioned above, CyCl treatment concomitantly inhibited NF-κB activation and increased Nrf2 translocation in colon cancer cells with or without TNF-α stimulation. To determine whether these events were casually related, Nrf2 expression was silenced by the transfection of cells with siRNA. As shown in Figure 7A, luciferase reporter assays showed that Nrf2 silencing directly affected the transcription of NF-κB. HCT116, HT29, and SW620 cells were treated with CyCl after transient transfection with an NF-κB promoter-luciferase reporter plasmid with or without co-transfection of Nrf2 siRNA. The experimental results showed that CyCl treatment decreased NF-κB reporter activity, as expected, and knockdown of Nrf2 abolished the inhibitory effect of CyCl on NF-κB transcriptional activity. Next, Western blot analysis showed that CyCl inhibited the phosphorylation of IκBα and IKKα/β in HCT116 cells transfected with control siRNA; however, these inhibitory effects of CyCl were not observed in HCT116 cells transfected with Nrf2 siRNA (Figure 7B), suggesting that CyCl inhibits NF-κB activation via Nrf2. Collectively, these results demonstrate that the anti-colon cancer activity of CyCl achieved by Nrf2 activation resulting in apoptosis is mediated by inhibition of the NF-κB signaling pathway. 

### 3.5. CyCl Inhibits Colon Cancer Foci Formation

**Figure 8:**
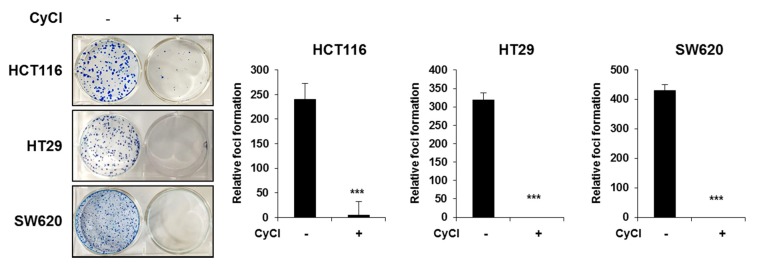
CyCl inhibits colon cancer foci formation. Colony formation assay shows a significant reduction in colony formation in HCT116, HT29, and SW620 cell lines treated with 50 µM of CyCl. Quantitative analyses are given in the form of bar graphs for HCT116, HT29, and SW620 cells, respectively. A dramatic decrease in colony formation is evident from the figure. Statistically significant values are marked with an asterisk. (*** *p* < 0.001).

Considering that the formation of foci in cancer cells in an important feature of carcinogenesis [27], we implemented the clonogenic assay to determine the capacity of CyCl in tumor colony formation. Three colon cancer cells were seeded at very low densities and their capacity to develop colonies was monitored for a period of 2 weeks. As shown in Figure 8, the formation of foci was almost completely blocked by CyCl treatment in HCT116, HT29, and SW620 cells. These results suggest that CyCl inhibits the malignant phenotype of these colon cancer cells.

## 4. Discussion

CRC is the third most diagnosed cancer in males worldwide and diverse risk factors, such as familial history, lifestyle, and environmental and genetic elements, can influence the development of CRC [1,2]. Several inflammatory cytokines and growth factors have also been reported to mediate colorectal carcinogenesis, and the severity of inflammation has been directly related to CRC risk [2]. Especially, TNF-α has been studied as one of the important factors involved in the pathogenesis of CRC [28]. TNF-α affects not only cell growth, differentiation, and function but also causes genetic mutations promoting cancer development, and modulates the expression of inflammatory genes [29,30,31]. Therefore, we used TNF-α stimulation to potentiate the malignant phenotype of colon cancer cells in this study. Most genes regulated by TNF-α have a site that binds to the transcription factor NF-κB [31]. A considerable amount of literature has shown that activation of the NF-κB signaling pathway may cause CRC [2], in which NF-κB acts as a key mediator of cellular proliferation and apoptosis [32]. In the present study, we evaluated the efficacy of CyCl on NF-κB signaling in TNF-α treated colon cancer cells. We found that CyCl treatment was able to inhibit the transcriptional activity, nuclear translocation, and upstream signaling of the NF-κB pathway as well as the activation of NF-κB-regulated genes. These results indicate that CyCl may effectively reduce the inflammation and proliferation associated with NF-κB signaling in CRC.

In connection with the emergence of chemoresistance, bioactive phytochemicals have attracted attention for the prevention and treatment of cancer. To find an effective natural compound that inhibits the NF-κB signaling pathway, IKKβ kinase activity was analyzed with an in-house natural product library, and CyCl was found as a potential candidate for reducing IKKβ phosphorylation. Recently, an anthocyanins-enriched extract from elderberries, in which five cyanidin-based anthocyanins were identified, inhibited proliferation and induced apoptosis in melanoma cells [33]. Cyanidin-3-*O*-glucoside, a cyanidin derivative, inhibited NF-κB signaling molecules and had a protective effect through activation of the Nrf2 pathway in intestinal epithelial cells exposed to TNF-α [34]. However, the anti-cancer function and molecular mechanism of CyCl in CRC have not yet been fully clarified. Our study has revealed that CyCl inhibited proliferation and induced apoptosis in HCT116, HT-29, and SW620 cells in a time- and concentration-dependent manner. We found that CyCl induced apoptosis through the activation of both intrinsic and extrinsic pathways by blocking the expression of inhibitors of apoptosis (IAP), such as XIAP, cIAP1, and cIAP2, and activation of caspase-3 and the cleavage of PARP via downregulation of NF-κB. It has been reported that the expression of XIAP, cIAP1, and cIAP2 is directly regulated by NF-κB activity [35]. Since the Bcl family proteins are important regulators of apoptosis, we also examined the effect of CyCl on the expression of Bcl2 and Bax. Gene expression analysis by qRT-PCR showed that treatment with CyCl decreased Bcl2 expression and increased Bax expression in colon cancer cells. Bcl2 has been reported to be overexpressed in colon cancer [36], and overexpression of Bcl2 or Bcl-xl suppresses apoptosis and promotes cell survival [37]. Moreover, the Bcl2-mediated inhibition of apoptosis restores the tumorigenicity of spontaneously regressive colon tumors in vivo [38]. The inhibition of Bcl2 expression and the increase in Bax level by CyCl thus provides a mechanistic basis of apoptosis induction by the compound in colon cancer cells. 

The potential role of ROS in carcinogenesis and disease progression has been studied for a long time [39]. Basal levels of ROS in cancer cells are higher than in normal cells due to increased metabolic activity and changes in cellular signaling [40]. In particular, the proliferating cells (stem or progenitor cells) in the colon have been shown to be very sensitive to the redox environment, thus excessive oxidative stress causes DNA damage, genomic instability, and abnormal gene regulation, all of which are associated with colorectal carcinogenesis [39,41]. Among the signaling pathways responsible for defense against oxidative stress, the Nrf2 signaling pathway is considered of great importance [42]. Nrf2 is a key transcription factor controlling cellular homeostasis in response to oxidative stress, which is involved in the regulation of the cellular redox balance, antioxidant, and detoxification responses [7,42]. Therefore, Nrf2 activation provides cytoprotection against numerous insults, including oxidative and toxic agents, various chronic diseases, and even cancer initiation [43]. In addition, Nrf2 activation has been reported to confer cellular protection by reducing excessive tumor growth and drug resistance in cancer cells [44]. In fact, Nrf2-knockout mice showed increased inflammation, colitis-associated aberrant crypt foci, and colitis-associated CRC [45,46,47]. Our results showed that CyCl treatment activated the antioxidant signaling pathway regulated by Nrf2. Treatment with CyCl induced Nrf2 expression, its translocation into the nucleus, and its binding to the ARE promoter, as well as the subsequent expression of Nrf2-regulated genes (HO-1 and NQO-1).

Nrf2 has been regarded as a cytoprotective factor through the regulation of defense mechanisms; however, there is increasing evidence that Nrf2 activation may not be beneficial for all cancer types and stages [48]. Indeed, Nrf2 activation in tumors protects cancer cells from excessive oxidative stress or chemotherapy, creating a favorable environment for the survival of cancer cells [48]. Interestingly, our results showed that CyCl induced apoptosis through the activation of Nrf2 signaling in colon cancer cells, suggesting that activation of the Nrf2 signaling pathway by CyCl promotes colon cancer cell apoptosis. This apoptotic mechanism triggered by CyCl in colon cancer cells is in contradiction with the well-known protective effect of Nrf2 that promotes the survival of normal and cancerous cells [10,44]. A possible mechanism to explain the apoptosis by CyCl is the interaction of Nrf2 with NF-κB. Recent studies suggest that there is functional crosstalk between the Nrf2 pathway and NF-κB signaling [25]. The absence of Nrf2 can exacerbate NF-κB activity, while NF-κB can regulate Nrf2 activity, having both positive and negative effects on target gene expression [49,50]. The lack of Nrf2 has been associated with augmented cytokine production, more pronounced NF-κB activity, and a neurodegenerative phenotype in Nrf2 knockout mice [51,52]. In addition, Thimmulappa and colleagues demonstrated enhanced IKKβ activity in Nrf2^−/−^MEFs, augmenting the phosphorylation of IκBα and its subsequent degradation [53]. Here, Nrf2 activation and NF-κB inhibition were strongly observed in CyCl-treated colon cancer cells and these results correlate with increased apoptosis and decreased colony formation in these cells. Moreover, knockdown of Nrf2 abrogated apoptosis and the inhibition of NF-κB signaling in CyCl-treated colon cancer cells. These findings suggest that the anti-colon cancer effect of CyCl is mediated by Nrf2 activation. The detailed mechanisms for the crosstalk between Nrf2 and NF-κB by CyCl need further investigation.

## 5. Conclusions

In conclusion, the present study showed that the activation of the Nrf2 signaling pathway by CyCl can reduce proliferation and colony formation in colon cancer cells by inhibiting the NF-κB signaling pathway and inducing apoptosis. To our knowledge, we have described for the first time the involvement of Nrf2 in the apoptotic response mediated by NF-κB inhibition. Therefore, CyCl may be a potential therapeutic strategy for treating CRC through the modulation of Nrf2 signaling.

## Figures and Tables

**Table 1:** List of antibodies.

Antibody	Dilution	Product No.	Species of Origin and Supplier
β-actin	1:4000	sc-47778	Mouse polyclonal, Santa Cruz Biotechnology, Inc.
α-tubulin	1:1000	sc-5286	Rabbit polyclonal, Santa Cruz Biotechnology, Inc.
LaminB	1:1000	sc-6217	Goat polyclonal, Santa Cruz Biotechnology, Inc.
PARP	1:1000	9542	Rabbit polyclonal, Cell Signaling Technology, Inc.
c-caspase3	1:1000	9661L	Rabbit polyclonal, Cell Signaling Technology, Inc.
Bax	1:1000	sc-7480	Mouse polyclonal, Santa Cruz Biotechnology, Inc.
XIAP	1:1000	sc-11426	Rabbit polyclonal, Santa Cruz Biotechnology, Inc.
p-IκBα	1:1000	2856	Rabbit polyclonal, Cell Signaling Technology, Inc.
IκBα	1:1000	9242	Rabbit polyclonal, Cell Signaling Technology, Inc.
p-IKKα/β	1:1000	2697	Rabbit polyclonal, Cell Signaling Technology, Inc.
IKKα	1:1000	2682	Rabbit polyclonal, Cell Signaling Technology, Inc.
IKKβ	1:1000	2678	Rabbit polyclonal, Cell Signaling Technology, Inc.
P65	1:1000	8242	Rabbit polyclonal, Cell Signaling Technology, Inc.
P50	1:1000	sc-1190	Goat polyclonal, Santa Cruz Biotechnology, Inc.
NQO1	1:1000	ab34173	Mouse polyclonal, Abcam, Inc.
HO-1	1:2000	ADI-SPA-895	Rabbit polyclonal, Enzo, Inc.
Nrf2	1:1000	ab62352	Rabbit polyclonal, Abcam, Inc.

**Table 2:** List of primers.

	Accession Number	Gene	Forward	Reverse	bp
qRT PCR	NM138763	Bax	CCT GTG CAC CAA GGT GCC GGA ACT	CCA CCC TGG TCT TGG ATC CAG CCC	99
	NM000657	Bcl2	TTG TGG CCT TCT TTG AGT TCG GTG	GGT GCC GGT TCA GGT ACT CAG TCA	114
	NM001166	cIAP1	GCTTCCGTGTTTGCTGCG	ACTGCAGGGGGACAAAATAGG	78
	NM001165	cIAP2	TCTGGGCAGCAGGTTTACAA	CCCGAGATTAGACTAAGTCCCTT	127
	NM020529	NFκbia	TGT GCT TCG AGT GAC TGA CC	TCA CCC CAC ATC ACT GAA CG	81
	NM032721	NFκbid	ACC CCA TCT GTG AAT GAG GC	GTC CAG AAC CCA CAG TCT CC	73
	NM000584	IL-8	TCC TTG TTC CAC TGT GCC TTG	TGC TTC CAC ATG TCC TCA CAA	101
	NM000600	IL-6	AGG GCT CTT CGG CAA ATG TA	GAA GGA ATG CCC ATT AAC AAC AA	62
	NM000594	TNF-α	GAA AGT CCC GTG GAA ATC CC	CTG CGC AGT CTG CTT TGC	70
	NM006164	Nrf2	AGGTTGCCCACATTCCCAAA	AGTGACTGAAACGTAGCCGA	118
	NM000903	NQO1	AAG AGC ACT GAT CGT ACT GG	CTT CAG TTT ACC TGT GAT GTC C	172
	NM002133	HO-1	CAA CAT CCA GCT CTT TGA GG	AGA AAG CTG AGT GTA AGG AC	204
	NR003286	18s rRNA	GCAATTATTCCCCATGAACG	GGCCTCACTAAACCATCCAA	111
RT PCR	NM001166	c-IAP1	GCCTTTCTCCAAACCCTCTT	ATTCGAGCTGCATGTGTCTG	355
	NM182962	c-IAP2	GCAGGCAAAGGCTAGTCATC	GAATGCTGCACAGAGACCAA	370
	NM006164	Nrf2	CGG TATGCA ACAGGACAT TG	ACTGGTTGGGGTCTTCTGTG	280
	NR003286	18s rRNA	CCCAACTTCTTAGAGGGACAAGT	TAGTCAAGTTCGACCGTCTTCTC	350

## Supplementary Materials

The following are available online at https://www.mdpi.com/2076-3921/9/4/285/s1, Figure S1: CyCl induces apoptosis in colon cancer cell lines, Figure S2: CyCl suppresses the NF-κB signaling pathway in colon cancer cells, Figure S3: CyCl induces Nrf2 activation and the expression of antioxidant enzymes, Figure S4: Knockdown of Nrf2 by Nrf2 siRNA abolishes CyCl-induced apoptosis.
