# Supplementary material for: Cyanidin Chloride Induces Apoptosis by Inhibiting NF-κB Signaling through Activation of Nrf2 in Colorectal Cancer Cells
Source: Antioxidants (Basel). 2020 Mar 27;9(4):285. doi: 10.3390/antiox9040285 (PMC7222181; doi:10.3390/antiox9040285)
Supplement: Supplementary file 1 [file antioxidants-09-00285-s001.pdf]

antioxidants

MDPI

Supplementary Figures

Cyanidin chloride induces apoptosis by inhibiting NF-κB signaling through activation of Nrf2 in colorectal cancer cells

Da-Young Lee [1], Sun-Mi Yun [1], Moon-Young Song [1], Ki-Won Jung [1] and Eun-Hee Kim [1,*]

[1] College of Pharmacy and Institute of Pharmaceutical Sciences, CHA University, Seongnam, 13488, Korea; angela8804@naver.com (D.Y.L); sun21mi@naver.com (S.M.Y); wso219@naver.com (M.Y.S); pharmj@cha.ac.kr (K.W.J)

* Correspondence: ehkim@cha.ac.kr (E.H.K.); Tel.: +82-31-881-7179

Received: date; Accepted: date; Published: date

Abstract: Colorectal cancer (CRC) is the third most common cancer worldwide and a leading cause of cancer-related deaths in developed countries. Anthocyanins are a class of flavonoids, widely distributed in food, exhibiting important biological effects. Cyanidin chloride (CyCl) is the common type of anthocyanin with antioxidative and anti-inflammatory potential. The present study aimed to investigate the molecular mechanisms underlying the chemotherapeutic effects of CyCl in colorectal cancer cells. We found that CyCl treatment induced apoptosis as well as significant inhibition of cellular proliferation and colony formation in three colon cancer HCT116, HT29 and SW620 cells. In addition, CyCl suppressed nuclear factor-kappa B (NF-κB) signaling and induced the activation of the nuclear factor erythroid 2-related factor 2 (Nrf2) pathway in TNF-α-stimulated HCT116 cells. Nrf2 and NF-κB are two key transcription factors regulating antioxidative responses and cellular proliferation, respectively. In this study, knockdown of Nrf2 by siRNA transfection inhibited the effect of CyCl on NF-κB signaling and apoptosis, suggesting that there is a functional crosstalk between Nrf2 and NF-κB. Our findings demonstrate the important role of Nrf2 in inducing apoptosis through the involvement of NF-κB signaling in colorectal cancer cells, suggest that CyCl may be used as a potential therapeutic agent for CRC.

Keywords: cyanidin chloride; Nrf2; NF-κB; apoptosis; colorectal cancer

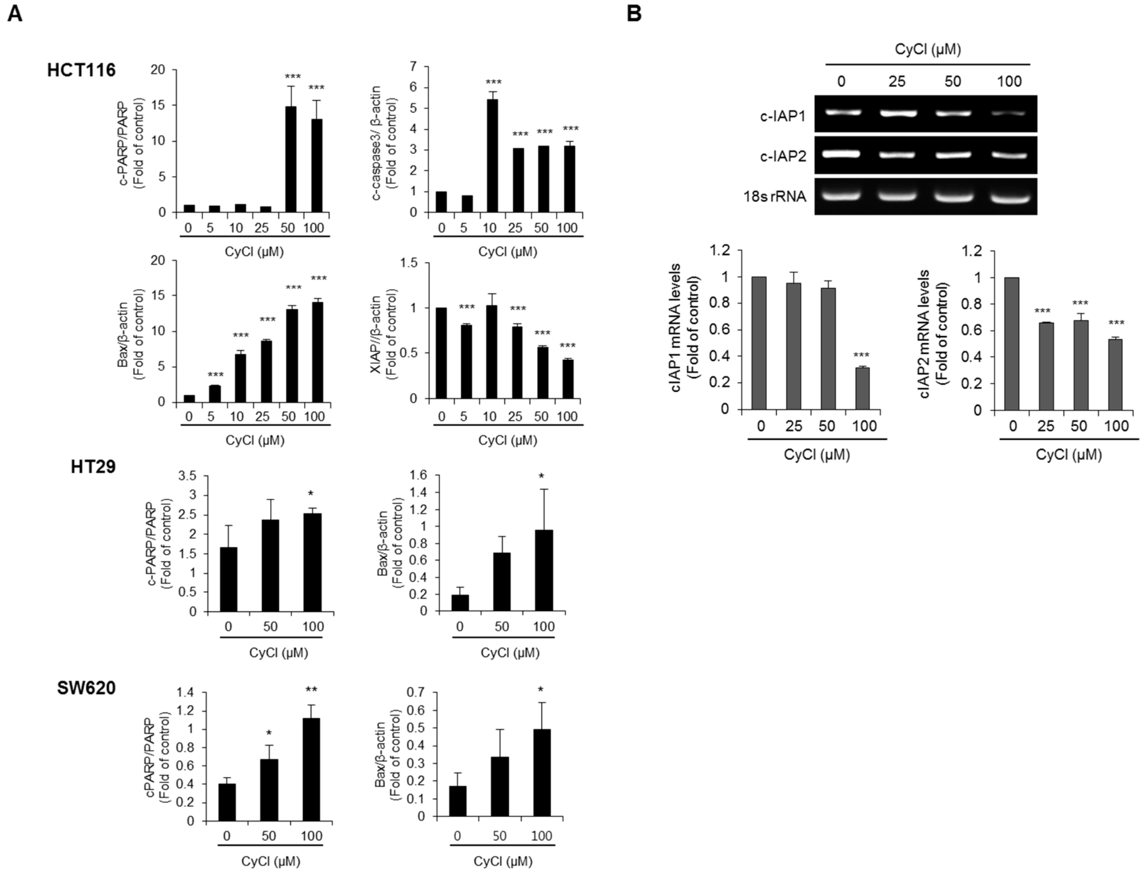

Figure S1. CyCl induces apoptosis in colon cancer cell lines. (A) HCT116, HT29, and SW620 cells were treated with indicated concentrations of CyCl and incubated for 24 h. Protein extracts were separated by SDS-PAGE, and Western blot analysis was conducted for the levels of Bax, XIAP, cleaved caspase-3 and PARP cleavage. Signal intensities of protein expression in Figure 3A were normalized to PARP or β-actin levels and are shown as a bar graph. (B) HCT116 cells were treated with 0, 25, 50 and 100 μM of CyCl and incubated for 24 h. The mRNA expression of c-IAP1 and c-IAP2 was examined by RT-PCR. The mRNA levels of c-IAP1 and c-IAP2 were normalized and are presented relative to change in relation to the control as a bar graph. All experiments were carried out in triplicate. *$P < 0.05$, **$P < 0.01$, and ***$P < 0.001$, significantly different compared with control.

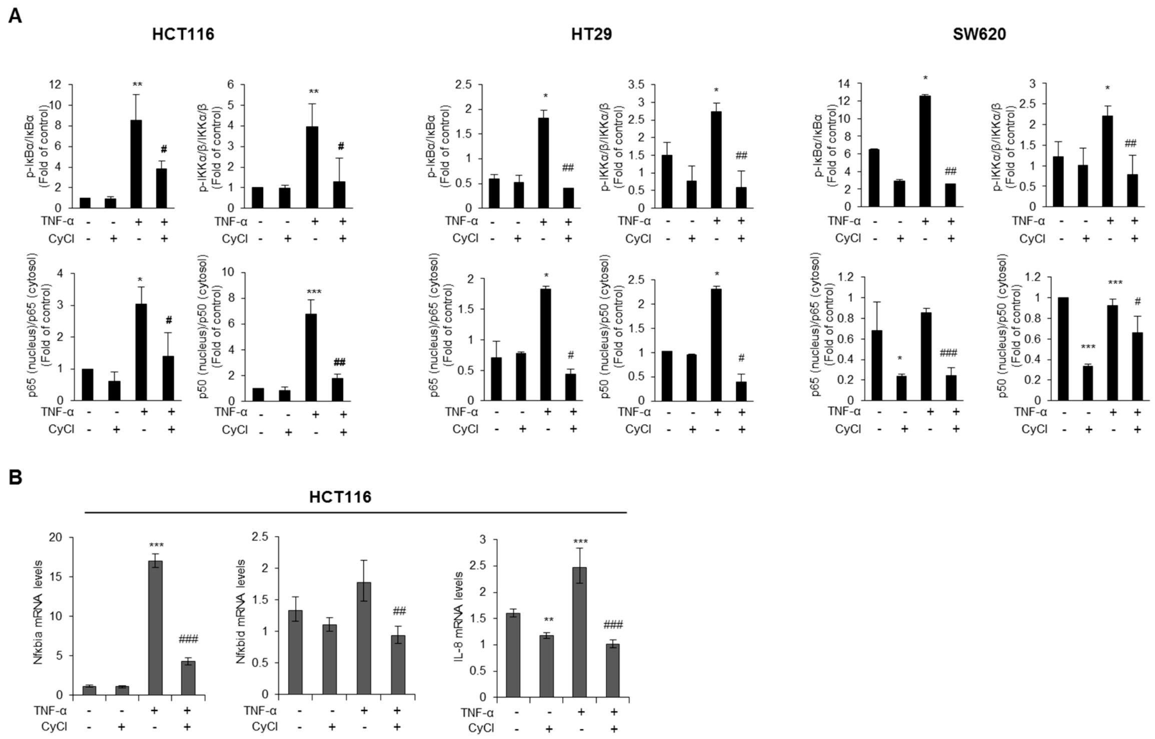

Figure S2. CyCl suppresses NF-κB signaling pathway in colon cancer cells. (A) HCT116, HT29, and SW620 cells were treated with TNF-α and 50 μM of CyCl and incubated for 24 h. Protein extracts were separated by SDS-PAGE, and Western blot analysis was conducted for the expressions of p-IκBα, IκBα, p-IKKα/β, IKKα, IKKβ, p65 and p50 protein. Signal intensities of protein expression in Figure 4B were normalized and are shown as a bar graph. (B) HCT116 cells were treated with TNF-α and 50 μM of CyCl and incubated for 24 h. RNA was extracted from the cells and mRNA expression of Nfκbia, NFκbid, and IL-8 was measured by qRT-PCR analysis. All experiments were carried out in triplicate. **P* < 0.05, ***P* < 0.01, and ****P* < 0.001, significantly different compared with control; #*P* < 0.05, ##*P* < 0.01, and ### *P* < 0.001, significantly different compared with TNF-α-treated cells.

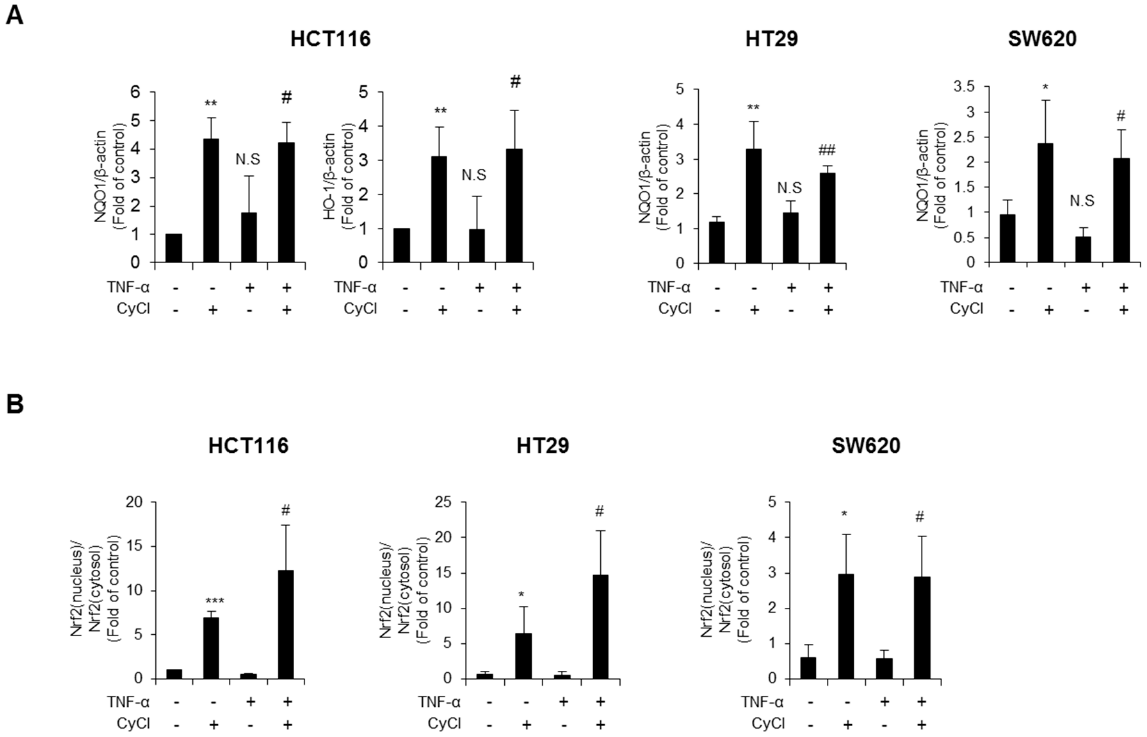

Figure S3. CyCl induces Nrf2 activation and the expression of antioxidant enzymes. (A) HCT116, HT29, and SW620 cells were treated with TNF-α and 50 μM of CyCl and incubated for 24 h. Protein extracts were separated by SDS-PAGE and Western blot analysis was conducted for the expression of NQO1 and HO-1 protein. Signal intensities of protein expression in Figure 5C were normalized and are shown as a bar graph. (B) HCT116, HT29, and SW620 cells were treated with TNF-α and 50 μM of CyCl and incubated for 24 h. After isolation of nuclear and cytosolic proteins, Western blot analysis was conducted for the changes in the subcellular localization of Nrf2. The quantitative results are expressed as the mean ± SD of three independent experiments. All experiments were carried out in triplicate. *$P < 0.05$, **$P < 0.01$ and ***$P < 0.001$, significantly different compared with control; #$P < 0.05$ and ##$P < 0.01$, significantly different compared with TNF-α-treated cells.

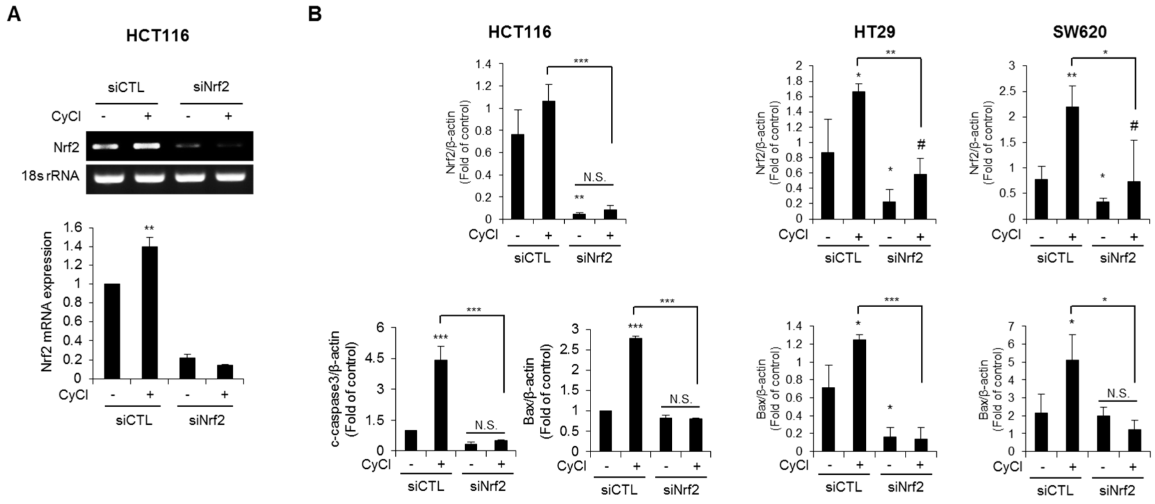

Figure S4. Knockdown of Nrf2 by Nrf2 siRNA abolishes CyCl-induced apoptosis. (A) HCT116 cells were transfected with 15 nM of control or Nrf2 siRNA. After 24 h, cells were treated with 50 μM of CyCl for 24 h and harvested. The expression of Nrf2 mRNA was measured by RT-PCR. (B) HCT116, HT29, and SW620 cells were transfected with 15 nM of control or Nrf2 siRNA for 24 h and treated with 50 μM of CyCl for 24 h. After protein isolation, Western blot analysis was conducted for the

levels of cleaved caspase-3, Bax and Nrf2 protein. Signal intensities of protein expression in Figure 6B were normalized and are shown as a bar graph. All experiments were carried out in triplicate. $^{*}P < 0.05$, $^{**}P < 0.01$ and $^{***}P < 0.001$, significantly different compared with empty vector transfected control; $^{\#}P < 0.05$, significantly different compared with siNrf2 transfected control.

© 2020 by the authors. Submitted for possible open access publication under the terms and conditions of the Creative Commons Attribution (CC BY) license (http://creativecommons.org/licenses/by/4.0/).
